# Addressing challenges in children’s mental health in disaster-affected areas in Japan and the Philippines – highlights of the training program by the National Center for Global Health and Medicine

**DOI:** 10.1186/s12919-018-0159-0

**Published:** 2018-12-19

**Authors:** Masahide Usami, Marian Fe Theresa Lomboy, Naoko Satake, Crystal Amiel M. Estrada, Mitsuya Kodama, Ernesto R. Gregorio Jr, Yuriko Suzuki, Ramir B. Uytico, Minerva P. Molon, Ikuhiro Harada, Keita Yamamoto, Kumi Inazaki, Hirokage Ushijima, Cynthia Leynes, Jun Kobayashi, Romeo R. Quizon, Tatsuro Hayakawa

**Affiliations:** 1 Department of Child and Adolescent Psychiatry, Kohnodai Hospital, National Center for Global Health and Medicine, Ichikawa, Japan; 2 College of Public Health, University of the Philippines Manila, Manila, Philippines; 3 Department of Psychiatry, National Center Hospital of Neurology and Psychiatry, Kodaira, Japan; 4 School of Health Sciences, Faculty of Medicine, University of the Ryukyus, Nakagusuku, Okinawa, Japan; 5 Department of Adult Mental Health, Institute of Mental Health, National Center of Neurology and Psychiatry, Kodaira, Japan; 6 Department of Education, Regional Office VIII, Leyte, Philippines; 7 Department of Health, Regional Office VIII, Leyte, Philippines; 8 Office of Social Work Service, Kohnodai Hospital, National Center for Global Health and Medicine, Ichikawa, Japan; 9 Department of Psychiatry, Philippine General Hospital, University of the Philippines Manila, Manila, Philippines; 10 Department of Psychiatry, Kohondai Hospital, National Center for Global Health and Medicine, Ichikawa, Japan

**Keywords:** Child, Mental health, Training program, Disaster, Japan, The Philippines, Community-based support network

## Abstract

**Background and purpose:**

Natural disasters such as earthquakes, typhoons, floods, and volcanic eruptions frequently occur in Republic of Philippines and mental health care for children affected by these natural disasters is a major public health concern. Aiming to train health professionals on children’s mental health, to conduct a situational analysis to identify the local needs and resources for children’s mental health, and to propose a mental health program for children that can be transferred from Japan to the Philippines, the National Center for Global Health and Medicine (NCGM) conducted a training program for children’s mental health in disaster-affected areas in Japan and the Philippines in June, October, and December, 2017. The training was organized by NCGM for the Program for International Promotion of Japan’s Healthcare Technologies and Services funded by Ministry of Health, Labour, & Welfare, Japan in relation to the Memorandum of Understanding in the Field of Healthcare between NCGM in Japan and University of the Philippines Manila, College of Public Health.

**Key highlights:**

The training program consisted of classroom trainings, site visits, and round table discussions in Japan and the Philippines. The classroom trainings and site visits focused on two points: the experiences of individuals and families who survived the Great East Japan Earthquake (GEJE) in 2011 and super typhoon Haiyan in 2013 and the program and activities, especially on mental health, of various government and non-government organizations in helping the affected families and communities. The round table discussion, on the other hand, was conducted to identify challenges related to children’s mental health in disaster-affected areas and to develop recommendations to address these challenges.

The major recommendations for the Philippines were to give equal emphasis to physical and psychosocial preparedness and to develop a comprehensive program to care for carers. In Japan, public health and mental health should be integrated in the Disaster Medical Service. Experts from both countries should also generate evidence on the effectiveness of interventions in reducing mental health stigma and collaborate with school personnel and communities in order to learn more about psychosocial preparedness. Finally, mental health must be mainstreamed in programs not only in Japan but also in other countries.

**Implications:**

The training program enabled key stakeholders to describe the current situation of mental health in Japan and the Philippines, to identify mental health challenges common to disaster-affected areas in both countries, and to propose short- and long-term plans and recommendations. The training program is expected to address the mental health needs of children in disaster-affected areas through a responsive community-based support network. The training participants agreed to form a network and build partnerships toward the common goal of mainstreaming community-based support for children’s mental health in disaster-affected areas in Japan and the Philippines.

## Background

Mental health is an emerging priority in the field of global health because mental disorders continue to contribute a substantial disease burden to low- and middle-income countries. Disasters affect the physical, psychological, social, and economic aspects of an individual, family, and community. In populations affected by disasters, the prevalence of mental health problems can be two to three times higher than that of the general population [1–4]. In Asia, the incidence of disaster-related post-traumatic stress disorder (PTSD) and general PTSD symptoms ranged from 8.0 to 37.0% [5]. In the Philippines, after super typhoon Haiyan affected the Visayas Region, approximately 800,000 people suffered from mental health problems. Of this number, about 10% or 80,000 people were dealing with severe depression [6].

Children, among other groups, are vulnerable to the psychological impacts of disasters and the impact can vary depending on the severity of the disaster. According to the American Academy of Pediatrics, approximately 25% of children who are exposed to a disaster have experienced symptoms of post-traumatic stress disorder (PTSD), anxiety, depression, and panic attack. Children often differ in their reactions to disasters because they are still in the process of developing emotional, cognitive, behavioral, and sensory skills. Moreover, they lack the experiences and coping skills that would help them face the traumatic consequences of disasters [3]. They usually depend on their families when dealing with the traumatic impacts of disasters. When compared to adults, children are more likely to suffer from mental health problems like depressive and post-traumatic symptoms, because they worry that a disastrous event may happen again and that they may lose their loved ones [3, 5]. The traumatic effects of disasters to children can last long after the disaster has occurred. While previous studies have shown that children are vulnerable to the psychological impacts of disasters [3, 7–19], research has not been done in the Philippines to assess the level of psychological preparedness of schoolchildren. While the Philippine government recognizes the importance of mental and psychosocial support for victims of disasters, oftentimes help is given only after the disaster has occurred. These problems are often compounded by limitations in human resources who can provide psychosocial support at the community level.

In Japan, the Bureau of International Health Cooperation, National Center for Global Health and Medicine (NCGM) conducted projects on global extension of medical technologies. Experts in the field of health policy, social security, health care, and health industry were deployed to developing countries. Through these activities, Japan was able to share their medical institutions’ experiences related to the public health insurance scheme and to promote excellent medical technologies, drugs, and equipment. As part of these initiatives, the Department of Psychiatry and Child and Adolescent Psychiatry of Kohnodai Hospital, NCGM co-created a training program for children’s mental health in disaster-affected areas in the Philippines.

The main aim of this training program was to share to the Philippines the activities of various local government, non-government organizations, and NCGM to assist children and families affected by the Great East Japan Earthquake (GEJE) in Japan. Furthermore, the minor aim of this program was to discuss the development of a community mental health network to support children’s mental health in disaster-affected areas in the Philippines and Japan.

## Project description

The Philippines is one of the countries with the highest risks to disasters. All geographic areas in the country are vulnerable to climate hazards. Recently, issues regarding children’s mental health in disaster-affected areas have emerged in the Philippines. During the response phase after super typhoon Haiyan, some communities in the Philippines implemented various activities in line with disaster mental health. These included information dissemination, community mobilization, strengthening of community and family support, creation of safe spaces, inclusion of psychosocial support in education, psychological intervention, clinical management of mental disorders, and many other activities [20].

In Japan, mental health professionals employed a clinical approach to treat mental disorders among children affected by disasters [15, 21, 22]. It is believed that children have special needs different from adults, so whatever trauma they experienced during childhood can have lasting effects. Child psychiatrists used psychosocial care to promote children’s mental health [1, 4]. Linking education with mental health is vital as it promotes communication or coordination among schools, communities, and clinicians. Moreover, it is necessary to collaborate with the families, medical staff, government staff, and educational institutions in order to provide the best treatment to children with traumatic symptoms after devastating disasters. In support of these activities, NCGM conducted trainings for children’s mental health and site visits in the disaster-affected areas in Japan and the Philippines. A round table discussion was held to inform local supporters about children’s mental health in affected areas and to build a support network based on local needs.

## Project contexts

The training program consisted of two trainings in Japan and the Philippines and one round table discussion in Japan.

The first training was held in Ichikawa and Ishinomaki City, Japan from June 5 to 9, 2017 (Table 1). Participants from the Philippines, who came from training institutions and communities previously affected by disasters, attended the training and engaged in discussions with Japanese experts. The site visits were done in schools and disaster-affected areas in Ishinomaki City in Japan.

**Table 1:** Training at Ichikawa and Ishinomaki, Japan

	Title	Venue
Experiences in areas affected by the Great East Japan Earthquake	Disaster risk reduction and management experiences in theGreat East Japan Earthquake	Ichikawa, Japan
	Mental health care for the aftermath of the Great East Japan Earthquake	
	Disaster damages in Ishinomaki	
	Mental health care supports in Ishinomaki offered by Kohnodai Hospital	
	Situation of departments of child and adolescent psychiatry in Japan	
	Support activities and follow-up studies of children’s mental health in Ishinomaki by the Department of Child and Adolescent of Kohnodai Hospital	
	Child supporter conference in Ishinomaki	
	Support for the health and welfare section in the City of Ishinomaki	
	Japanese version of disaster guidebook for children with mental disorder	
	Disaster management in schools in the Philippines	
	Experiences of the representatives from Tacloban on disaster management	
	Disaster mental health/mental health management in the Philippines	
Experiences in areas affected by super typhoon Haiyan	Disaster management in schools in the Philippines	Ichikawa, Japan
	Experiences of the representatives from Tacloban on disaster management	
	Disaster mental health/mental health management in the Philippines	
Site Visit on Ishinomaki City	Watanoha Elementary School	Ishinomaki, Japan
	Watanoha Junior High School	
	Temporary housing, Restoration housing	
Workshops on Activities of the City of Ishinomaki	Education Board of the City of Ishinomaki	Ishinomaki, Japan
	From a stand point of a public health nurse of Health Promotion Division	
	From a stand point of a public health nurse of Parental Support Division	
	Activities of Child Abuse Protection Center	
	Activities of Life Restoration Support Division	
	Activities of Miyagi Disaster Mental Health Care Center	

The second training was held in Leyte, Philippines from October 23 to 27, 2017 (Table 2). Individuals and families who survived typhoon Haiyan shared their experiences. The activities of various government and non-government organizations in providing assistance, particularly mental health, to the affected families were also presented.

**Table 2:** Training at Leyte, Philippines

	Title	Venue
Lecture from Philippines	Presentation of the activities of the Child Protection Network and the Child Protection Unit- Philippine General Hospital for mental health	Manila, Philippines
Site Visit	Observation at the Department of Child and Adolescent Psychiatry, PGH	
Lecture from Japan	Background about NCGM, activities and plans during the visit to Tacloban City	Tacloban, Philippines
Lecture form Philippines	Mental Health Program for Disasters	
	The World Health Organization Mental Health Gap Action Program (MHGAP) in Region 8	
	Disaster Mental Health/MHPSS Activities of the Department of Social Welfare and Development	
	Disaster Mental Health Activities of the Save the Children	
	Disaster Mental Health Activities of Plan International	
Field visit and sharing of experiences during typhoon Yolanda and programs for mental health	Tanauan Elementary School	Tacloban, Philippines
	Permanent relocation site in Tanauan	
	San Jose National High School	
	Office of Civil Defense Region 8	
	Eastern Visayas Regional Medical Center/Child Protection Unit	
Group discussion	Small group discussion/meeting—mental health research	Manila, Philippines

The round table discussion (RTD) was held at the University of the Ryukyus, Okinawa, Japan on December 4 to 5, 2017 (Table 3). The main objectives of the RTD were to identify common challenges in disaster mental health and to discuss plans and recommendations to overcome these challenges.

**Table 3:** Round Table Discussion at Okinawa, Japan

	Title	Venue
Review of the training program	Review and Evaluation of the Training Program Part 1	Okinawa, Japan
	Review and Evaluation in the Training Program Part 2	
discussion	Round-table discussion Part 1	
	Round-table discussion Part 2	

## Participants

The National Center for Global Health and Medicine invited ten health experts from the College of Public Health and the Philippine General Hospital, University of the Philippines Manila, Department of Health Regional Office VIII, and Department of Education Regional Office VIII, and two researchers from the University of Ryukyus, Okinawa, Japan to attend the first training. The second training in the Philippines was attended by seven Japanese health experts (three psychiatrists, two child psychiatrists, one social worker, and one public health researcher) and 22 participants from Manila and Leyte. Finally, the RTD was attended by five heads of agencies from the Philippines and five Japanese mental health experts. The profile of participants from the Philippines and Japan are shown in Table 4. The discussions focused mainly on the GEJE and super typhoon Haiyan.

**Table 4:** Country and professions of participants

Country	Profession	Number
Philippines	Teaching	2
	Medical Officer	2
	Assistant Professor	3
	Psychologist	3
	Nurse	3
	Child Psychiatry	3
	Public Health Researcher	3
	Project Development Officer	3
	Physician	3
	Community Development Worker	1
	Social Worker	1
	Assistant Superintendent	1
	Teacher/Lawyer	1
Japan	Psychiatrist	3
	Child Psychiatrist	3
	Social worker	2
	Public health researcher	1
	Medical student	1

## Observations from field implementation

### Implementation of disaster risk reduction management (DRRM)

In the Philippines, DRRM activities focused mainly on physical preparedness like evacuation drills and procurement of life-saving equipment. There is little emphasis given to mental health preparedness activities like capacity building for psychological first aid (PFA) and the provision of mental health and psychosocial support (MHPSS) especially to children and other people affected by disasters. While the Philippine Department of Health (DOH) has implemented the mental health gap action program (mhGAP) to capacitate field health workers, continuity and sustainability is needed to ensure that preparedness efforts for mental health are proactive rather than reactive. Moreover, the question of whether the trainees are able to apply or have the confidence to apply what they learned from the mhGAP training is yet to be explored. In Japan, disaster preparedness activities have intensified after the GEJE of 2011. Mental health teams consisting of psychiatrists and social workers were organized and deployed to disaster-stricken areas. As of September 1, 2011, 57 teams were dispatched to work with local mental health professionals to deliver pre-disaster psychiatric services. They also provided psychoeducation at communal shelters [7]. Despite these, concrete preparedness plans which can be implemented at the community level are still lacking. The Japanese government also launched the Disaster Medical Assistance Team (DMAT) for emergency response but public health and mental health were not yet integrated in the response.

### Community based mental health and networking for mental health

Networking for disaster mental health is a serious issue in the Philippines. In Region VIII, the Department of Education’s network for mental health has not been established and its only connection with other government agencies is with the Department of Social Welfare and Development (DSWD). On the other hand, the Department of Health works with the Disaster Risk Reduction and Management Council (DRRMC) of various local government units in addressing health concerns. Mental health is given little priority at the local level probably due to stigma and other competing health priorities. In Japan, the government established mental health care centers called *kokoro no care* centers after the 2011 GEJE. However, these centers did not function fully because of the limited network that can facilitate coordination among key stakeholders.

### Community-based mental health care for children

Community-based mental health care is not recognized in the communities in the Philippines. Parents still depend on the efforts of the Department of Health and the Department of Education. There were attempts to start community-based mental health care in Region VIII but due to changes in administration, the initiatives were not sustained. At present, efforts are focused on autism. In the field of Japanese child psychiatry, many child mental health professionals focus on children with neurodevelopmental disorders and suicide and traumatic experiences. These children are usually treated through hospital-based care. There are also limitations in the number of child psychiatrists in the communities. This problem is further compounded by the lack of public health nurses in health centers.

### Caring for the mental health carers

In the Philippine Department of Education, there is still a need for activities and programs for teachers who respond to calamities and disasters. These programs should be carefully developed because the teachers serve as the front liners in times of disasters. The Department of Health, on the other hand, conducts regular program implementation review and gives recognition and appreciation to responders who are considered as unsung heroes. Debriefing of field workers is usually done by medical doctors, psychiatrists, and psychologists in DOH; however, psychological debriefing is not a standard practice in the context of disaster response [8]. In Japan, there is high concern for local carers especially during the recovery phase. The activities are limited to orientations, wrap up meetings, and appreciation ceremonies which are done during the response phase. In addition, mental health screening for health workers and teachers are being conducted. In the recovery phase, a more systematic approach is needed by embedding caring for carers in the occupational health program [9].

### Psychosocial preparedness in schools and communities

Psychosocial preparedness pertains to interventions that address both the social and psychological needs of an individual. Psychological support is beneficial to mental health as it promotes inner capacity building while social support is strengthened through social networks like family and community functions [10]. Based on field observations, there is minimal integration of psychosocial preparedness in the education curriculum in the Philippines. At present, psychosocial preparedness remains to be achieved. Schools and communities prepare physically but do not focus much on psychological preparedness. In Japan, the stakeholders are not aware of any trainings for psychosocial preparedness but there is some integration of disaster preparedness and mental health, such as avoidance of violence, into the curriculum. Although there is no specific curriculum on disaster mental health in Japan, a project by the Kohnodai team consisting of annual mental health check-ups, follow-up of the pupils, and support for the teachers serve to foster psychosocial preparedness. This is a secondary prevention program to detect high-risk children, and at the same time, it can be seen as a primary prevention program to raise awareness of mental health and psychosocial preparedness of children as well as teachers.

## Recommendations

### Implementation of disaster risk reduction and management

In the Philippines, psychosocial and physical preparedness should be given equal emphasis. The mhGAP training implemented by DOH with the World Health Organization (WHO) should be sustained and expanded to other agencies like the Department of Education and the Department of Social Welfare and Development. It is likewise important to get the commitment of the local government units (LGUs) and the Department of Interior and Local Government (DILG). In Japan, public health and mental health should be integrated in the DMAT and Disaster Psychiatric Assistance Team (DPAT). Finally, mental health must be integrated in programs not only in Japan but also in other countries.

### Community based mental health and networking for mental health

The network and partnership that has been established through the mental health training should be sustained. The network that has been formed will be very useful in addressing the mental health needs of children in disaster-affected areas. Systematic coordination between local and national agencies should be strengthened in both countries. There is a need to establish a coordination system and to clarify the implementation of children’s mental health programs at the municipal level. Likewise, dissemination of best practices for coordination and networking at the community level in Leyte and Ishinomaki should be done. Finally, it is important to have a deeper understanding of the regional family system and culture to help responders and experts cater to the needs of the community.

### Community-based mental health care for children

In the Philippines, there is a need to come up with a program involving the Department of Education and parents through the Parents-Teachers Association (PTA). The local chief executives (LCEs) should likewise be engaged to support the implementation of community-based mental health care by rural health care units and well-baby clinics. Moreover, there is a need for coordination among various offices offering mental health services. In Japan, local psychiatrists and pediatricians should be integrated into the communities.

### Caring for the mental health carers

There is a need to develop a comprehensive program for carers in the Philippines. In Japan, experts should teach stakeholders how to manage stress after a tremendous disaster. Experts also need to raise the awareness of policy makers and the general public on the experiences of carers. The mainstreaming of caring for the carers into the industrial and occupational health system should likewise be explored.

### Psychosocial preparedness in schools and communities

There is a need to integrate psychosocial preparedness in school and community activities in the Philippines. In Japan, mental health experts need to work with school personnel in order to learn more about psychosocial preparedness. Cooperation with the local community is indispensable for psychosocial preparedness.

## Conclusion

The training program enabled key stakeholders to describe the current situation of mental health in the Philippines and Japan, to identify mental health challenges common to disaster-affected areas in both countries, and to propose short- and long-term plans and recommendations. The training program is expected to address the mental health needs of children in disaster-affected areas through a responsive community-based support network. The training participants agreed to form a network and build partnerships toward the common goal of mainstreaming community-based support for children’s mental health in disaster-affected areas in Japan and the Philippines.

## Abbreviations

DILG: Department of Interior and Local Government
DMAT: Disaster Medical Assistance Team
DOH: Philippine Department of Health
DPAT: Disaster Psychiatric Assistance Team
DRRM: Disaster Risk Reduction Management
DRRMC: Disaster Risk Reduction and Management Council
DSWD: Department of Social Welfare and Development
GEJE: Great East Japan Earthquake
LCE: Local Chief Executives
LGU: Local Government Units
mhGAP: Mental Health Gap Action Program
MHPSS: Mental Health and Psychosocial Support
NCGM: National Center for Global Health and Medicine
PFA: Psychological First Aid
PTA: Parents-Teachers Association
PTSD: Post-traumatic stress disorder
RTD: Round table discussion
